# Comparison of Time Domain and Spectral Domain Optical Coherence Tomography in Measurement of Macular Thickness in Macular Edema Secondary to Diabetic Retinopathy and Retinal Vein Occlusion

**DOI:** 10.1155/2012/354783

**Published:** 2012-07-25

**Authors:** Elham Hatef, Afsheen Khwaja, Zubir Rentiya, Mohamed Ibrahim, Matthew Shulman, Peykan Turkcuoglu, Yasir Sepah, Jianmin Wang, Roomasa Channa, Millena Bittencourt, Abeer Akhtar, Jangwon Heo, Diana V. Do, Quan Dong Nguyen

**Affiliations:** Retinal Imaging Research and Reading Center (RIRRC), Wilmer Eye Institute, Johns Hopkins Hospital, 600 North Wolfe Street, Maumenee 745, Baltimore, MD 21287, USA

## Abstract

*Purpose*. To evaluate macular thickness, agreement, and intraclass repeatability in three optical coherence tomography (OCT) devices: the time domain (TD) Stratus OCT and two spectral domain (SD) OCTs, Spectralis and Cirrus SD-OCT, in eyes with macular edema secondary to diabetic retinopathy (DR) and retinal vein occlusion (VO). *Methods*. In a prospective observational study at a university-based retina practice, retinal thickness tomography was performed simultaneously for fifty-eight patients (91 eyes) with DR and VO employing a time domain and two spectral domain OCTs. Agreement in macular measurements was assessed by constructing Bland-Altman plots. Intraclass repeatability was assessed by intraclass correlation coefficients (ICCs). *Results*. Based on the Bland-Altman plots for central macular thickness, there was low agreement between the measurements of Cirrus SD-OCT and Stratus OCT, Spectralis OCT and Stratus OCT, as well as Spectralis OCT and Cirrus SD-OCT among DR and RVO patients. All three devices demonstrated high intraclass repeatability, with ICC of 98% for Stratus OCT, 97% for Cirrus SD-OCT, and 100% for Spectralis OCT among DR patients. The ICC was 97% for Stratus OCT, 79% for Cirrus SD-OCT, and 91% for Spectralis OCT among RVO patients. *Conclusion*. There are low agreements among interdevice measurements. However, intraclass repeatability is high in both TD and SD-OCT devices.

## 1. Introduction

Automated measurements of retinal thickness and volume using optical coherence tomography (OCT) and imaging-processing software are commonly used in the diagnosis and management of retinal diseases such as diabetic retinopathy and age-related macular degeneration [1–4]. Time domain Stratus OCT (Carl Zeiss Meditec, Inc., Dublin, CA, USA) is the most widely used device both in clinical and research settings. Stratus OCT acquires images at a rate of 400 axial scans per second with an axial resolution of 10 *μ*m, and an ability to generate measurements with high repeatability [1]. The new generation of OCT devices employing spectral domain technology provides an axial resolution of approximately 3 *μ*m and improves visualization of retinal normal anatomy as well as microstructural changes in the neurosensory layer [5–8]. There are some differences in the intrinsic software algorithms that each device applies to calculate retinal thicknesses. A thickness map is calculated based on the data collected from an array of A-scans distributed across the macula. The number of A-scans used in Stratus OCT is fewer and weighted more towards the center of the scan. The method to correct for this effect, as well as different anatomical landmarks that each device uses to specify the outer retinal boundary, contribute to a different absolute value for thickness measurement [1]. 

There are similar differences among various spectral domain OCTs. These differences highlight the importance of repeatability testing for each device. The evaluation of association among thickness measurements by different devices is also an important issue to consider prior to using them interchangeably in clinical and research setting. The repeatability of macular measurements by time domain Stratus OCT and spectral domain Cirrus SD-OCT (Carl Zeiss Meditec, Inc.) has been tested in the context of diabetic macular edema (DME) [1]. However, to our knowledge, the repeatability of macular measurements obtained by Spectralis HRA/OCT (Heidelberg Engineering, Inc., Heidelberg, Germany) has not been determined in patients with different maculopathies [9]. The goal of the index study is to evaluate the repeatability and agreement of macular measurements by time domain Stratus OCT versus spectral domain Spectralis OCT and Cirrus SD-OCT in two retinal vascular diseases, diabetic retinopathy and retinal vein occlusion. 

## 2. Materials and Methods

Patients who had macular edema secondary to diabetic retinopathy (DR) and retinal vein occlusion (RVO), which was confirmed with fundus photography as well as fluorescein angiography, were eligible to be enrolled in the study. The diagnosis of macular edema secondary to DR or RVO was made by the two retina specialists (QDN and DVD). Eyes with other conditions resulting in macular thickening such as epiretinal membrane, vitreal macular traction, and neovascular age-related macular degeneration were excluded. Research design and patient deidentification were Health Insurance Portability and Accountability Act (HIPAA) compliant. Institutional Review Board (IRB) approval from the Johns Hopkins Medical Institutions was obtained. 

All study subjects were scanned with each of the three OCT machines in random order on a single visit by a single operator. Macular thickness measurements of nine standard subfields were analyzed. The fast macular thickness map protocol was used to scan patients with the Stratus OCT. Within a scan time of 1.9 seconds, the device scans six evenly spaced 6 mm radial lines consisting of 128 A-scans per line that intersect at the fovea (total of 768 sampled points) [1]. The 512 × 128 scan pattern was applied to obtain scans with Cirrus SD-OCT. In a scan time of 2.4 seconds, the device scans a 6 × 6 mm area of the retina with 128 horizontal lines, each consisting of 512 A-scans per line (total of 65,536 sampled points) [1]. 

The Spectralis OCT has a transverse (in tissue) resolution of 14 microns (*μ*m) and an axial (in tissue) resolution of 3.9 *μ*m. With a speed of 40,000 A-scans per second, the device scans a 6 × 6 mm area (20 × 15 degrees) of the retina with 19 horizontal raster scans and 240 *μ*m between every two lines, each line consisting of 428 A-scans (total of 8,143 sampled points). The thickness values in between the lines are calculated using sophisticated interpolation algorithms. The device employs its speed to rescan each line several times and averages the data points acquired (this is only possible with the Spectralis OCT because it is able to track the lines and change the scan position according to the eye movements, automatically correcting for motion artifacts) (*information obtained from the Spectralis HRA/OCT Manual*). 

We obtained two high-quality scans with each device. Scans without artifacts caused by eye movement and papillary shadowing were selected as the high-quality scans on Spectralis OCT as well as Stratus OCT and Cirrus SD-OCT. We did not exclude any poor scans due to media opacity and those without clear delineation of the retina layers. Such strategy provided us data to compare scans taken by different devices on eyes of different characteristics—those that were and were not ideal to undergo high-quality scan. We compared the scan results on different devices based on the thickness of the macula. During OCT scanning, we used an intrinsic fixation target to center the macular grid, along with manual centering of the grid by the operator for those images for which the grid outside fovea was not allowed. 

To calculate retinal thickness, we defined an internal and external retinal layer position by using the intrinsic retinal segmentation algorithms in each device. All three devices used the protocol from the Age-Related Eye Disease Study (AREDS) [10] to average retinal thickness within nine retinal subfields in a 6 mm diameter circle centered on the fovea. Retinal subfields were reported as central, inner superior, inner nasal, inner inferior, inner temporal, outer superior, outer nasal, outer inferior, and outer temporal. For Cirrus SD-OCT and Spectralis OCT, when OCT device defined the retinal segmentation algorithms on an incorrect anatomical site, we corrected it manually and separately reported the values after correction.

To make pair-wise comparisons of nine subfield retinal measurements between each pair of two devices, we used the two-tailed paired *t*-test with Bonferroni adjustment for multiple comparisons. By constructing Bland-Altman plots, we assessed the agreement in macular measurements between each device [11]. Intraclass repeatability was assessed by intraclass correlation coefficients (ICCs) for each of the two measurements of each patient on each of the three devices. We calculated the coefficient of repeatability (CRW) to assess and compare the intrasession repeatability of the three devices. CRW is defined as 1.96 ∗ SW, where SW stands for the intrasession within-subject standard deviation [1]. 

## 3. Results

Fifty-eight patients (91 eyes) were included in this study: 28 patients (61 eyes; seven patients had more than one visit, and the eyes were scanned and considered independently at each visit) had macular edema secondary to DR in one or both eyes, and 30 patients (30 eyes) had macular edema from RVO in one eye. The age range among patients with DR was 38.3 to 81.9 years (median 69.5 years) and 15 (53.6%) of them were male. The age range among patients with RVO was 40.6 to 87.8 years (median 68.3 years) and 20 (66.7%) of them were male. Central retinal vein occlusion (CRVO) was diagnosed in 13 (43.3%) of the patients and the remaining were diagnosed with branch retinal vein occlusion (BRVO). 

### 3.1. OCT Characteristics among DR Patients

**Figure 1:**
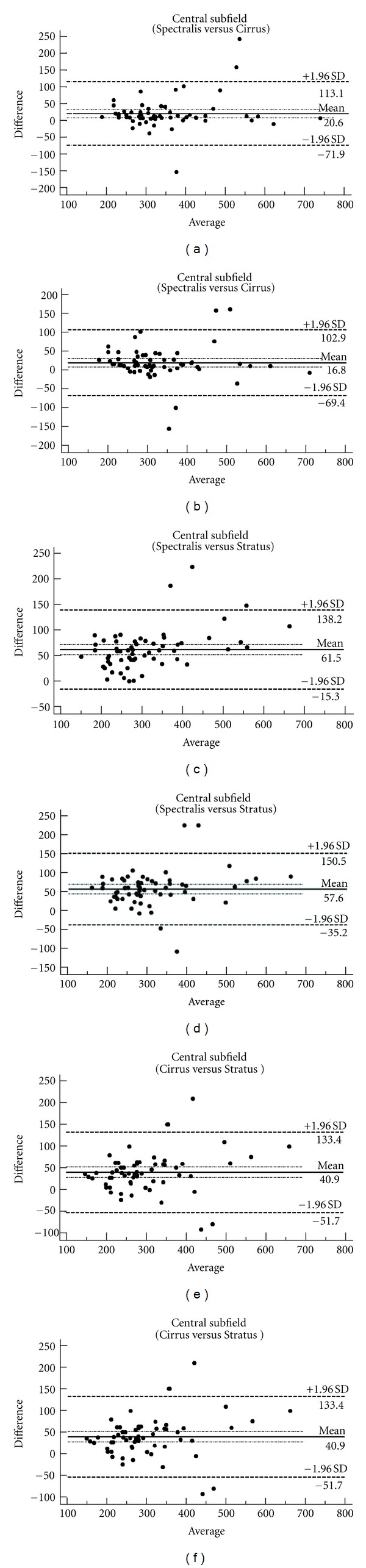
Bland-Altman plots of central subfield macular thickness between each device for the values before manual correction of algorithms (a, c, and e) and after manual correction (b, d, and f) in eyes with diabetic retinopathy. Solid lines: average mean difference; dotted lines: 95% confidence limits of agreement.

The mean central macular thickness was 289 *μ*m (standard deviation [SD]: 99 *μ*m) on Stratus OCT device, 323 *μ*m (SD: 108 *μ*m) on Cirrus SD-OCT and 344 *μ*m (SD: 116 *μ*m) on Spectralis OCT. These values changed to 322 *μ*m (SD: 108 *μ*m) on Cirrus SD-OCT and 340 *μ*m (SD: 108 *μ*m) on Spectralis OCT after manual correction of algorithm.  Table 1 presents the mean values and SDs for nine subfields on the three OCT devices as well as after-correction values for Cirrus SD-OCT and Spectralis OCT devices. The mean central macular thickness value was 21 *μ*m (95% confidence interval (CI): 9, 33 *μ*m) higher on Spectralis OCT compared to Cirrus SD-OCT and 61 *μ*m (95% CI: 51, 72 *μ*m) higher on Spectralis OCT compared to Stratus OCT. The differences decreased to 17 *μ*m (95% CI: 5, 28 *μ*m) between Spectralis OCT and Cirrus SD-OCT and to 58 *μ*m (95% CI: 45, 70 *μ*m) between Spectralis OCT and Stratus OCT values after manual correction of algorithms. The mean central macular thickness was 41 *μ*m (95% CI: 29, 53 *μ*m) higher on Cirrus SD-OCT compared to Stratus OCT. The difference was the same (41 *μ*m, 95% CI: 28, 53 *μ*m) after manual correction of algorithms.  Table 2 shows the mean difference and 95% CIs between retinal thickness values on the three OCT devices for nine different subfields before and after manual correction of algorithms. The Bland-Altman plot showed a poor agreement for central macular thickness measurements between each device before and after manual correction of algorithms (Figure 1). There was an ICC of 98% (95% CI: 97, 99%) among two measurements of central thickness values for each eye on the Stratus OCT device. ICC was reported as 97% (95% CI: 96, 99%) on Cirrus SD-OCT and 100% (95% CI: 99, 100%) on Spectralis OCT for central thickness values before manual correction of algorithms and 97% (95% CI: 96, 99%) on Cirrus SD-OCT and 99% (95% CI: 99, 100%) on Spectralis OCT for central thickness values after correction.  Table 3 shows the ICC for retinal thickness values on the three OCT devices for nine different subfields. For Cirrus SD-OCT and Spectralis OCT values, ICC is reported after manual correction of algorithms as well. For central thickness values on Stratus OCT, CRW was reported as 27 *μ*m. CRW was 36 *μ*m before and after manual correction of algorithms on Cirrus SD-OCT and 5 *μ*m before and 18 *μ*m after manual correction of algorithms on Spectralis OCT.  Table 4 presents CRWs for retinal thickness values on the three OCT devices for nine different subfields. For Cirrus SD-OCT and Spectralis OCT values, CRW is also reported after manual correction of algorithms. 

### 3.2. OCT Characteristics among RVO Patients

**Figure 2:**
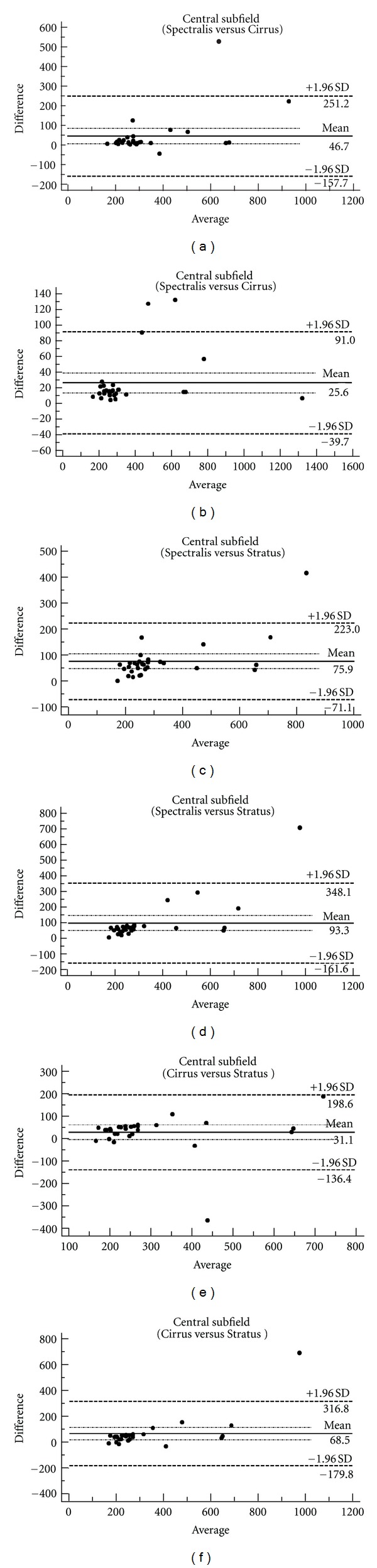
Bland-Altman plots of central subfield macular thickness between each device for the values before manual correction of algorithms (a, c, and e) and after manual correction (b, d, and f) in eyes with vein occlusion. Solid lines: average mean difference; dotted lines: 95% confidence limits of agreement.

The mean central macular thickness was 283 *μ*m (SD: 149 *μ*m) on the Stratus OCT device, 315 *μ*m (SD: 156 *μ*m) on Cirrus SD-OCT, and 359 *μ*m (SD: 198 *μ*m) on Spectralis OCT. The value changed to 353 *μ*m (SD: 241 *μ*m) on Cirrus SD-OCT and 376 *μ*m (SD: 245 *μ*m) on Spectralis OCT after manual correction of algorithm (Table 1). The mean central macular thickness value was 47 *μ*m (95% CI: 7, 86 *μ*m) higher on Spectralis OCT compared to Cirrus SD-OCT, and 76 *μ*m (95% CI: 48, 104 *μ*m) higher on Spectralis OCT compared to Stratus OCT. The difference decreased to 26 *μ*m (95% CI: 13, 38 *μ*m) between Spectralis OCT and Cirrus SD-OCT and 93 *μ*m (95% CI: 45, 142 *μ*m) between Spectralis OCT and Stratus OCT values after manual correction of algorithms. The mean central macular thickness was 31 *μ*m (CI: 1, 64 *μ*m) higher on Cirrus SD-OCT compared to Stratus OCT. The difference increased to 68 *μ*m (95% CI: 20, 117 *μ*m) after manual correction of algorithms (Table 2). There was poor agreement for central macular thickness measurements between each device before and after manual correction of algorithms (Figure 2). There was an ICC of 97% (95% CI: 95, 99%) among two measurements of central thickness values for each eye on the Stratus OCT device. ICC was reported as 79% (95% CI: 66, 93%) on Cirrus SD-OCT and 91% (95% CI: 84, 97%) on Spectralis OCT for central thickness values before manual correction of algorithms, and 99% (95% CI: 99, 100%) on Cirrus SD-OCT and 100% (95% CI: 100, 100%) on Spectralis OCT for central thickness values after correction (Table 3). For central thickness values on Stratus OCT, CRW was reported as 54 *μ*m. CRW was 146 *μ*m before and 30 *μ*m after manual correction of algorithms on Cirrus SD-OCT and 119 *μ*m before and 7 *μ*m after manual correction of algorithms on Spectralis OCT (Table 4).

## 4. Discussion

Our study is one of the inaugural investigations comparing the OCT findings in patients with macular edema from DR and RVO between a time domain OCT, Stratus OCT, and two spectral domain OCTs, Spectralis OCT and Cirrus SD-OCT [1, 9]. The SD OCTs reported higher values for macular thickness in all nine standard subfields in DR and RVO patients compared to TD Stratus OCT. The values obtained from the two spectral domain OCTs remained higher after manual correction of algorithms. Spectralis OCT detected higher macular thickness in all nine standard subfields compared to Cirrus SD-OCT. The same difference was detected by Forooghian et al. [1] comparing Cirrus SD-OCT to Stratus OCT, as well as by Lammer et al. [9] comparing Spectralis OCT, Cirrus SD-OCT, and Stratus OCT in patients with DME. 

The discrepancy among the three devices in terms of macular thickness values reflects differences in defining retinal segmentation algorithms. While Stratus OCT measures the thickness of the retina as the distance between the inner limiting membrane (ILM) and junction of the outer segment (OS) and inner segment (IS) of the photoreceptors, Cirrus SD-OCT reports it as the distance from the anterior border of the retinal pigment epithelium (RPE) to the ILM [1], while Spectralis OCT measures the distance from the posterior border of the RPE to the ILM. Therefore, the macular measurements are larger on Spectralis OCT compared to Cirrus SD-OCT and Stratus OCT. The mean difference between macular thickness measurements obtained by the Cirrus SD OCT and Stratus OCT in nine different subfields was close to 50 *μ*m in most of the subfields, which is the length of the outer segment of human photoreceptors [1, 12]. Bland and Altman invented the method of evaluating agreement between measurements of two devices by plotting their difference against their mean. The measurements can be used interchangeably when the 95% CI of agreement is within a clinically acceptable range [11]. Figures 1 and 2 present the Bland-Altman plots for the central subfield values on different devices. The 95% CIs for agreement between each two machines in DR and VO patients were very wide. The 95% CIs remained very large after manual correction of the algorithms. Therefore, macular thickness measurements obtained with each of the three devices cannot be used interchangeably with the measurements from the other device due to poor agreement among devices [1, 9]. 

We evaluated the repeatability of the macular thickness measurements of each device by performing two series of images with each device on each patient. The ICC was reported high for all the nine subfields on all three devices. ICC remained high after manual correction of algorithms for measurements by Cirrus SD-OCT and Spectralis OCT. Although different methods were applied to measure the repeatability of each OCT device, our results were consistent with other reports on Stratus OCT [13, 14], as well as Cirrus SD-OCT [1] and Spectralis OCT [9] for patients with DR. 

Thus far, there have not been any published reports on the repeatability of measurements of macular edema in RVO. ICC was reported less than 90% on Stratus OCT, Cirrus SD-OCT, and Spectralis OCT for some of the segments (81–100% among DR patients and 79–100% among those with RVO) before and after manual correction of algorithms. The low reported ICC could be due to chance. There is no physiological or anatomical basis to explain the difference in macular thickness measurements in these segments compared to the rest of the scanned area [1]. Since the 95% CI for all these measurements is wide and greater than 90%, the low ICC could be due to some outliers in the measurements for ICC. 

The CRW was reported as less than 40 *μ*m for Stratus OCT in DR patients and less than 55 *μ*m in RVO patients. Such index presents the half-width for the 95% CI in the thickness measurement variation from one OCT measurement to another for each patient. Our finding is similar to the Diabetic Retinopathy Clinical Research Network report on Stratus OCT in DME patients [2]. The CRW ranged from 11 *μ*m to 52 *μ*m for Cirrus SD-OCT in DR patients before and after manual correction of algorithms. It ranged from 10 *μ*m to 146 *μ*m before correction to 10 *μ*m to 49 *μ*m after correction of algorithms in RVO patients. The wide range of CRW before manual correction of algorithms reflects the inability of the device to recognize anatomical landmarks and define algorithms in the presence of extensive macular edema. The CRW was large for patients with ≥800 *μ*m in macular thickness, for which Cirrus SD-OCT was not able to recognize anatomical landmarks. The CRW range decreased dramatically after manual correction of algorithms. 

The CRW ranged from 4 *μ*m to 20 *μ*m for Spectralis SD in DR patients before and from 7 *μ*m to 39 *μ*m after manual correction of algorithms. It ranged from 9 *μ*m to 119 *μ*m before correction to 7 *μ*m to 28 *μ*m after correction of algorithms in RVO patients. The same problem of not being able to recognize anatomical landmarks and define algorithms in the presence of extensive amount of macular edema was detected in Spectralis OCT. The CRW range decreased dramatically after manual correction of algorithms. 

The current study provides valuable information regarding the performance of different OCT devices in macular edema from two different types of retinal vascular diseases. To the best of our knowledge, our OCT analysis in DR patients is one of the few reports with a large sample size on the comparison of SD OCTs among each other as well as with the TD OCT. Our analysis in RVO patients is the first report of SD OCTs in patients with RVO. The index study has evaluated the performance of OCT devices in a real-time clinic without omission of impaired images due to inability of the device to recognize anatomical landmarks. Unlike other reports on this subject, we also reviewed all images taken with SD OCTs on different types of patients and ocular and media condition, and made manual corrections of algorithms when necessary. Therefore, the index study also presents the performance of each device in actual, clinical situation where there are protean factors that may affect quality of scans. 

## 5. Conclusions

Although each OCT device has a unique method of defining algorithms and cannot be used interchangeably, each device has the ability to measure the retinal thickness accurately and repeatedly. The advantage of one SD OCT over the other or to a TD OCT might be in its ability to provide images with more details of different retinal layers and detect small changes in the normal anatomy of retina. Additional studies to evaluate the quality of images by each device to define subtle anatomical changes in different layers of retina are necessary to make informed decision to employ which of the available OCTs in a specific clinical setting. 

## Figures and Tables

**Table 1:** Mean ± standard deviation of nine standard subfields of three OCT devices.

	Diabetic macular edema					Vein occlusion				
		Before correction		After correction			Before correction		After correction	
	Stratus OCT	Cirrus SD-OCT	Spectralis OCT	Cirrus SD-OCT	Spectralis OCT	Stratus OCT	Cirrus SD-OCT	Spectralis OCT	Cirrus SD-OCT	Spectralis OCT
Central	282 ± 99	323 ± 108	344 ± 116	323 ± 108	340 ± 108	283 ± 149	315 ± 156	359 ± 198	353 ± 241	376 ± 245
Inner superior	299 ± 65	340 ± 60	363 ± 66	339 ± 60	358 ± 67	302 ± 100	357 ± 111	389 ± 161	377 ± 162	390 ± 168
Inner nasal	290 ± 58	336 ± 63	357 ± 68	336 ± 62	359 ± 72	319 ± 118	348 ± 87	389 ± 139	380 ± 167	397 ± 158
Inner inferior	280 ± 73	325 ± 80	346 ± 83	324 ± 80	343 ± 77	307 ± 110	327 ± 61	360 ± 101	366 ± 147	378 ± 144
Inner temporal	295 ± 83	343 ± 74	364 ± 82	343 ± 73	351 ± 76	300 ± 117	340 ± 97	364 ± 146	363 ± 175	381 ± 178
Outer superior	251 ± 36	289 ± 44	319 ± 42	289 ± 44	317 ± 40	258 ± 76	299 ± 69	345 ± 104	311 ± 79	331 ± 83
Outer nasal	261 ± 57	305 ± 66	319 ± 50	304 ± 66	323 ± 68	275 ± 60	301 ± 58	336 ± 67	327 ± 86	341 ± 82
Outer inferior	239 ± 84	283 ± 97	295 ± 34	325 ± 80	305 ± 87	258 ± 88	272 ± 51	329 ± 109	291 ± 63	326 ± 97
Outer temporal	253 ± 55	290 ± 53	314 ± 61	290 ± 53	306 ± 61	242 ± 74	277 ± 44	300 ± 76	287 ± 78	298 ± 74

**Table 2:** Mean difference between two OCT devices and 95% confidence interval of nine standard subfields.

	Diabetic macular edema						Vein occlusion					
	Before correction			After correction			Before correction			After correction		
	Spectralis OCT versus Cirrus SD-OCT	Cirrus SD-OCT versus Stratus OCT	Spectralis OCT versus Stratus OCT	Spectralis OCT versus Cirrus SD-OCT	Cirrus SD-OCT versus Stratus OCT	Spectralis OCT versus Stratus OCT	Spectralis OCT versus Cirrus SD-OCT	Cirrus SD-OCT versus Stratus OCT	Spectralis OCT versus Stratus OCT	Spectralis OCT versus Cirrus SD-OCT	Cirrus SD-OCT versus Stratus OCT	Spectralis OCT versus Stratus OCT
Central	21 [9, 33]	41 [29,53]	61 [51,72]	17 [5,28]	41 [29,53]	58 [45,70]	47 [7,86]	31 [1,64]	76 [48,104]	26 [13,38]	68 [20,117]	93 [45,142]
Inner superior	25 [11, 39]	40 [26,54]	65 [58,72]	18 [5,32]	40 [26,54]	59 [48,69]	31 [14,76]	55 [41,70]	84 [50,118]	15 [3,33]	75 [44,107]	90 [53,125]
Inner nasal	21 [9, 33]	46 [39,54]	67 [57,77]	23 [11,34]	46 [38,54]	69 [59,79]	44 [7,81]	26 [4,56]	70 [59,80]	20 [3,38]	58 [36, 81]	78 [58,97]
Inner inferior	21 [14, 28]	45 [36,53]	66 [58,74]	18 [12,24]	45 [36,53]	63 [54,72]	34 [6,75]	18 [22,58]	53 [43,63]	15 [3,26]	56 [22,90]	71 [42,99]
Inner temporal	21 [13, 29]	47 [36,59]	68 [60,77]	8 [3,19]	47 [36,59]	56 [40,71]	25 [12,61]	41 [20,61]	65 [46,83]	19 [8,31]	63 [35,93]	82 [50,114]
Outer superior	26 [14, 38]	38 [29,47]	68 [60,75]	26 [15,37]	38 [29,47]	64 [57,70]	43 [5,81]	41 [12,69]	79 [50,107]	22 [7,35]	52 [40,65]	72 [61,83]
Outer nasal	13 [6, 19]	43 [39,48]	56 [52,60]	18 [10,26]	43 [39,48]	61 [53,68]	34 [1,67]	25 [6,56]	59 [54,64]	15 [4,26]	51 [40,63]	66 [55,77]
Outer inferior	21 [12, 30]	44 [37,52]	64 [58,69]	26 [10, 42]	44 [36,51]	61 [56,66]	43 [12,97]	25 [12,61]	59 [48,70]	19 [13,25]	44 [37, 50]	62 [53,72]
Outer temporal	21 [13, 30]	38 [30,46]	63 [55,70]	11 [2, 20]	86 [70,101]	51 [40,62]	19 [0,37]	38 [16,60]	58 [47,69]	11 [6,16]	48 [37,58]	56 [50,62]

**Table 3:** Intraclass correlation coefficient and 95% confidence interval for repeated measures of each subfield by each OCT device.

Diabetic macular edema						Vein occlusion				
	Before correction		Stratus OCT	After correction		Before correction		Stratus OCT	After correction	
	Cirrus SD-OCT	Spectralis OCT		Cirrus SD-OCT	Spectralis OCT	Cirrus SD-OCT	Spectralis OCT		Cirrus SD-OCT	Spectralis OCT
	ICC^a^ (%) [95% CI^b^]	ICC (%) [95% CI]	ICC (%) [95% CI]	ICC (%) [95% CI]	ICC (%) [95% CI]	ICC (%) [95% CI]	ICC (%) [95% CI]	ICC (%) [95% CI]	ICC (%) [95% CI]	ICC (%) [95% CI]
Central	97 [0.96, 0.99]	100 [0.99, 1.00]	98 [0.97, 0.99]	97 [0.96, 0.99]	99 [0.99, 1.00]	79 [0.66, 0.93]	91 [0.84, 0.97]	97 [0.95, 0.99]	99 [0.99,1.00]	100 [1.00, 1.00]
Inner superior	82 [0.74, 0.90]	100 [0.99, 1.00]	93 [0.90, 0.97]	82 [0.74, 0.90]	100 [0.99, 1.00]	93 [0.89, 0.98]	100 [0.99, 1.00]	97 [0.95, 0.99]	99 [0.98, 1.00]	99 [0.99,1.00]
Inner nasal	98 [0.97, 0.99]	100 [0.99,1.00]	95 [0.92, 0.98]	98 [0.97, 0.99]	99 [0.99, 1.00]	89 [0.81,0.97]	83 [0.71, 0.95]	99 [0.99,1.00]	98 [0.96, 0.99]	100 [1.00, 1.00]
Inner inferior	96 [0.94, 0.98]	100 [0.99, 1.00]	97 [0.96, 0.99]	96 [0.94, 0.98]	100 [0.99, 1.00]	93 [0.89, 0.98]	95 [0.92, 0.99]	97 [0.95, 0.99]	99 [0.98, 1.00]	99 [0.99, 1.00]
Inner temporal	94 [0.92, 0.97]	100 [0.99, 1.00]	98 [0.97, 0.99]	94 [0.92, 0.97]	98 [0.97, 0.99]	97 [0.95, 0.99]	98 [0.96, 0.99]	96 [0.93, 0.99]	99 [0.99, 1.00]	100 [0.99, 1.00]
Outer superior	81 [0.72, 0.90]	99 [0.99, 1.00]	90 [0.84, 0.95]	80 [0.72, 0.90]	93 [0.89, 0.97]	98 [0.96, 0.99]	87 [0.76, 0.97]	96 [0.94, 0.99]	99 [0.98,1.00]	99 [0.98, 1.00]
Outer nasal	99 [0.99, 1.00]	100 [0.99, 1.00]	97 [0.95, 0.98]	99 [0.99, 1.00]	95 [0.93, 0.98]	98 [0.97, 0.99]	98 [0.96, 0.99]	94 [0.90, 0.99]	97 [0.94, 0.99]	100 [1.00, 1.00]
Outer inferior	98 [0.98, 0.99]	98 [0.97, 0.99]	98 [0.96, 0.99]	98 [0.98, 0.99]	100 [0.99, 1.00]	99 [0.98, 1.00]	99 [0.99, 1.00]	97 [0.95, 0.99]	99 [0.99,1.00]	99 [0.99, 1.00]
Outer temporal	90 [0.85, 0.95]	97 [0.96, 0.99]	88 [0.82, 0.94]	90 [0.85, 0.95]	89 [0.83, 0.94]	91 [0.84, 0.97]	100 [0.99, 1.00]	91 [0.84, 0.98]	98 [0.97, 1.00]	100 [0.99, 1.00]

^a^ICC: intraclass correlation coefficient.

^b^CI: confidence interval.

**Table 4:** Coefficient of repeatability (*μ*m) for each subfield in each OCT device.

	Diabetic macular edema					Vein occlusion				
	Cirrus SD-OCT		Stratus OCT	Spectralis OCT		Cirrus SD-OCT		Stratus OCT	Spectralis OCT	
	Before correction	After correction		Before correction	After correction	Before correction	After correction		Before correction	After correction
Central	36	36	27	5	18	146	30	54	119	7
Inner superior	52	52	33	9	8	54	30	33	18	28
Inner nasal	18	18	26	6	14	60	49	19	110	11
Inner inferior	31	30	24	8	7	30	31	37	48	24
Inner temporal	35	35	22	7	20	33	25	50	41	20
Outer superior	36	36	23	6	20	20	15	27	64	19
Outer nasal	12	11	21	4	29	16	30	34	19	8
Outer inferior	24	23	26	10	8	10	10	31	15	11
Outer temporal	34	34	40	20	39	32	19	55	9	11
